# Meso- and Macro-Mechanical Analysis of the Frost-Heaving Effect of Void Water on Asphalt Pavement

**DOI:** 10.3390/ma15020414

**Published:** 2022-01-06

**Authors:** Jiancun Fu, Aiqin Shen

**Affiliations:** 1 School of Highway, Chang’an University, Xi’an 710064, China; saq6305@163.com; 2 Shandong Transportation Institute, Jinan 250000, China

**Keywords:** asphalt mixture, water phase transition, frost-heaving effect, thermal-mechanical coupling, numerical simulation

## Abstract

In cold regions, many types of structural damages are caused by the frost heaving of asphalt pavements. Hence, it is important to quantitatively determine the frost-heaving effect of asphalt pavement using a mechanical method to control frost-heaving damage. In this study, first, the internal voids of the asphalt mixture were regarded as a single void, and the water phase transition generating the freezing water in the voids was simulated using a simplified hollow sphere model to create a uniform internal pressure. Second, the prediction equation of the equivalent linear expansion coefficient was proposed by taking the phase transition of water in the saturated asphalt mixture voids into account. A step function was used during the phase transition of water to determine the sudden change in the equivalent linear expansion coefficient, heat capacity, density, and thermal conductivity. Finally, the typical cooling conditions were simulated with the water phase transition and the nonwater phase transition. The experimental results showed that the proposed model could accurately simulate the effect of frost heaving. Higher stress and strain were generated on the surface and in the interior of the pavement, and the positions of maximum stress and strain occurred on the pavement surface under the frost-heaving conditions. The compressive strength of the asphalt mixture in a uniaxial compression test is about 4.5–6 MPa with a single freeze–thaw cycle. Furthermore, when frost heaving occurs on the asphalt pavement between 5.8 and 6.5 MPa, the numerical simulation method can be used to calculate the internal stress of the structure, which found that the compressive stress under the frost-heaving condition was the same magnitude as the compressive strength under the freeze–thaw testing condition.

## 1. Introduction

The defects caused by the freeze–thaw cycle of an asphalt mixture in pavements are typical in cold regions [1,2]. When the temperature is lower than 0 °C, the freezing water in the internal voids expands the volume of the mixture and separates the skeleton in the asphalt mixture. Eventually, repeated freeze–thaw action leads to the loosening of the asphalt mixture and further results in pavement defects [3,4].

A large number of laboratory findings showed that the freeze–thaw cycle significantly reduced the strength, fatigue life, and durability of different asphalt mixtures [5,6,7,8,9,10,11]. The technology of CT (Computed Tomography) scanning is used to study the freeze–thaw damage process of the asphalt mixture, which can effectively reveal the meso characteristics of the asphalt mixture during the freeze–thaw process in comparison with the general testing methods for mix design [12,13]. The results of CT show that frost heaving can lead to the expansion of a single void, the merging of two separate voids, and the formation of new voids, which can increase the size and number of voids and cause significant attenuation of asphalt mixture performance [14,15,16].

Based on the above understanding, describing the frost-heaving effect through a mechanical model has become a research hotspot because of the frost-heaving problem. The main mechanical models include the macroscopic damage model based on experimental results [17,18] and the mesoscopic mechanical model considering void characteristics [19,20,21]. The former can quantitatively describe the performance degradation of the asphalt mixture, which is caused by the frost-heaving effect; however, it is difficult to analyze the influence of different mesoscopic characteristics on the frost-heaving effect of asphalt mixture, although the latter considers the size and distribution characteristics of internal voids, the modeling process is complex, and the calculation efficiency is low. Additionally, it is hard to directly introduce it into the calculation of full-scale pavement structure to analyze the frost-heaving effect of pavement structures so that the influence of frost heaving on the macroscale cannot be captured.

Therefore, establishing a numerical analysis method of the frost-heaving effect of asphalt mixture and asphalt pavement structure can consider the void characteristics of the mixture, has higher computational efficiency, and has become a key problem to be solved. In this paper, the simulation method for the frost-heaving effect of the asphalt mixture was simplified utilizing a macro–meso combination, and the numerical simulation of the frost-heaving effect of asphalt mixture and pavement structure was effectively realized. Firstly, the frost-heaving of asphalt mixture was simplified to the problem of ice sphere expansion constrained by a skeleton from the meso-level perspective, and then the equivalent linear expansion coefficient model corresponding to the frost heaving of asphalt mixture was derived. The model was introduced into the elastic finite element analysis of full-scale asphalt pavement structure; on this basis, the influence of frost heaving on the pavement structure was analyzed. Finally, the numerical simulation results were verified from both qualitative and quantitative perspectives; this proves the reliability and rationality of the mechanical model and analysis methods established in this paper.

## 2. Methods

### 2.1. Meso-Mechanical Model

In the early research on this topic, much effort has been put into studying the frost resistance of cement concrete. It is commonly believed that the pores of the concrete are made up of macropores and micropores, and the minimum order of magnitude of the pores is a few nanometers. The water inside the pore does not freeze even at very low temperatures as the pore radius of concrete is less than a certain value, according to the Laplace equation [22,23,24,25]. However, many studies have shown that the voids in asphalt mixtures are mesoscopic with a magnitude grade of a few millimeters, and nonicing voids do not exist [26].

**Figure 1:**
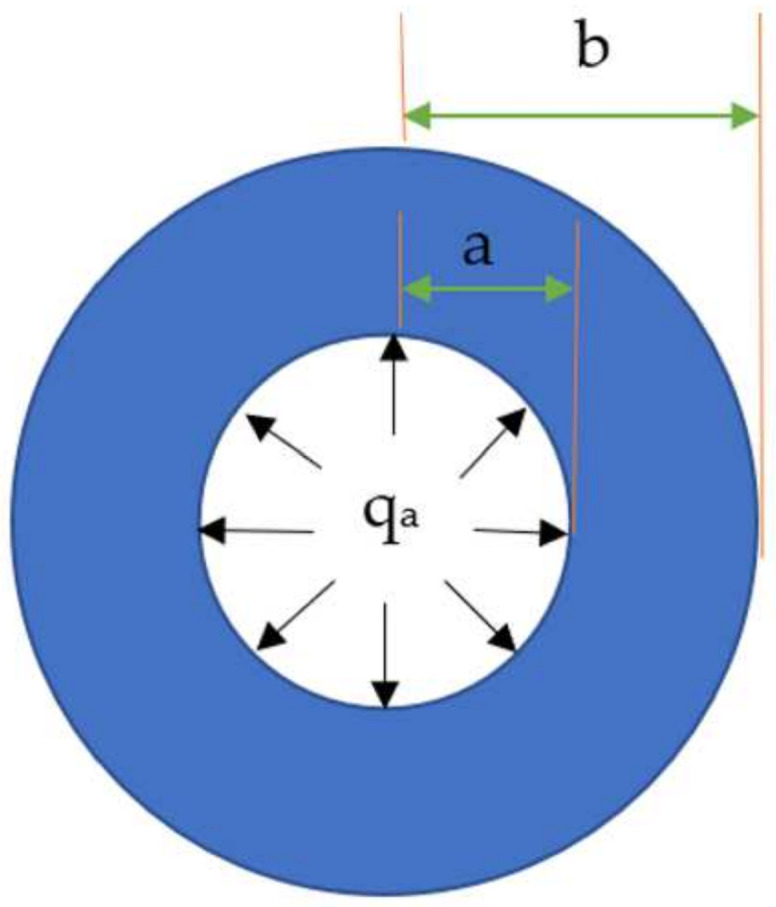
Meso-mechanical model of the asphalt mixture frost heaving.

In this study, it is argued that when the temperature is lower than 0 °C, the asphalt mixture will expand due to the freezing of void water (the interstitial water studied in this paper only considers the water in the gap between asphalt mixture frameworks, and the whole article does not include other void water). At the meso level, the void water is regarded as a solid sphere according to the force characteristics of void water freezing in the asphalt mixture, and the asphalt mixture skeleton is regarded as a hollow sphere; the phase change process of void water freezing becomes a process of a sphere expansion under spherical constraint, as shown in Figure 1 [27]. The skeleton constrains the icing expansion of water in the asphalt mixture voids, and the volume expansion force, generated by the ice expansion, uniformly acts on the inner surface of the asphalt mixture skeleton. As shown in Figure 1, the volume expansion force induced by water icing is equivalent to the uniformly distributed load, q_a_, and a and b denote the inner radius of the void and the outer radius of the asphalt mixture skeleton, respectively. The volumetric expansion coefficient of water icing is 1.09. The void radius increases by approximately 0.03a according to the conversion relationship between the volume and radius. In fact, water icing expansion is constrained by the skeleton, which assumes that the radial compression is ∆b and the actual ice expansion is 0.03a − ∆b.

### 2.2. Establishing the Equation for the Equivalent Linear Expansion Coefficient

The radial displacement of a hollow sphere (mixture skeleton) subjected to internal pressure, q_a_, and external pressure, q_b_, is expressed as follows [28]:

(1) $$u_{1}=\frac{\left(1+\mu \right)r}{E}\left[\frac{\frac{b^{3}}{2r^{3}}+\frac{1-2\mu }{1+\mu }}{\frac{b^{3}}{a^{3}}-1}q_{a}-\frac{\frac{a^{3}}{2r^{3}}+\frac{1-2\mu }{1+\mu }}{1-\frac{a^{3}}{b^{3}}}q_{b}\right]$$

where *E* is the elastic modulus of the skeleton, Pa; µ is the Poisson’s ratio of the skeleton; and *r* is the radius of the skeleton, m.

For a solid sphere (ice), a→0, at this time, q_a_ does not exist; that is, q_a_ = 0. Therefore, when the external pressure is q_b_, the radial displacement of the solid sphere can be expressed as

(2) $$u_{1}=-\frac{\left(1-2\mu_{i}\right)q_{b}r}{E_{i}}$$

where *E*_i_ is the elastic modulus of ice, Pa; and µ_i_ is the Poisson’s ratio of ice.

For ice, when *r* = 1.03a, u_1_ = ∆b, and q_b_ = q. Substituting these values in Equation (2), we obtain

(3) $$-\Delta b=-\frac{1.03a\left(1-2\mu_{i}\right)q}{E_{i}}$$

For a hollow sphere subjected to internal pressure q_a_, its radial displacement is given by

(4) $$u_{2}=\frac{\left(1+\mu \right)r}{E}\left[\frac{\frac{b^{3}}{2r^{3}}+\frac{1-2\mu }{1+\mu }}{\frac{a^{3}}{b^{3}}-1}q_{a}\right]$$

For a hollow sphere, when *r* = a, u_2_ = 0.03a − ∆b, and q_a_ = q. Substituting these values in Equation (4), we obtain

(5) $$\frac{\left(1+\mu \right)a}{E}\left[\frac{\frac{b^{3}}{2a^{3}}+\frac{1-2\mu }{1+\mu }}{\frac{a^{3}}{b^{3}}-1}q\right]=0.03a-\Delta b$$

By rearranging Equation (5), the expression for ∆b obtained is as follows:

(6) $$\Delta b=\frac{0.03aE}{\left(1+\mu \right)a\left[\frac{\frac{b^{3}}{2a^{3}}+\frac{1-2\mu }{1+\mu }}{\frac{b^{3}}{a^{3}}-1}\times \frac{E_{i}}{1.03a\left(1-2\mu_{i}\right)}\right]+E}$$

The expansion displacement of the ice sphere is given by

(7) $$\Delta u=0.03a-\frac{0.03aE}{\left(1+\mu \right)a\left[\frac{\frac{b^{3}}{2a^{3}}+\frac{1-2\mu }{1+\mu }}{\frac{b^{3}}{a^{3}}-1}\times \frac{E_{i}}{1.03a\left(1-2\mu_{i}\right)}\right]+E}$$

While performing the numerical simulation, a temperature interval of (T_S_, T_E_) is generally assumed in the phase transition process of water during its freezing, and the deformation is caused by the phase transition of water in the asphalt mixture, which can be transformed into a general temperature deformation. The corresponding equivalent linear expansion coefficient is expressed as:

(8) $$\gamma_{wi}=\frac{\Delta u}{b\times (T_{S}-T_{E})}$$

where γ_wi_ is the equivalent linear expansion coefficient of the asphalt mixture during the water phase transition, T_S_ is the temperature at the beginning of the water phase transition, and T_E_ is the temperature at the end of the water phase transition.

The general temperature strain is calculated as follows:

(9) $$\varepsilon_{th}=\frac{\Delta u}{b}=\gamma (T)\times \left(T-T_{ref}\right)$$

where ε_th_ is the temperature strain; γ(T) is the linear expansion coefficient, °C; and T_ref_ is the reference temperature, °C.

The frost-heaving strain is equivalent to the temperature strain. After introducing the water phase transition temperature interval, the total temperature strain of the asphalt mixture, including the frost-heaving strain, can be expressed by the following step function:

(10) $$\varepsilon_{th}=\left\{\begin{array}{ll} \gamma_{w}\left(T-T_{ref}\right) & T>T_{S}\\ \gamma_{w}\left(T_{S}-T_{ref}\right)+\gamma_{wi}\left(T-T_{S}\right) & T_{E}<T<T_{S}\\ \gamma_{wi}\left(T_{S}-T_{E}\right)+\gamma_{i}\left(T-T_{ref}\right) & T<T_{E} \end{array}\right.$$

### 2.3. Analysis of the Response of the Structural Mechanics

#### 2.3.1. Establishment of the Full-Scale Model

**Figure 2:**
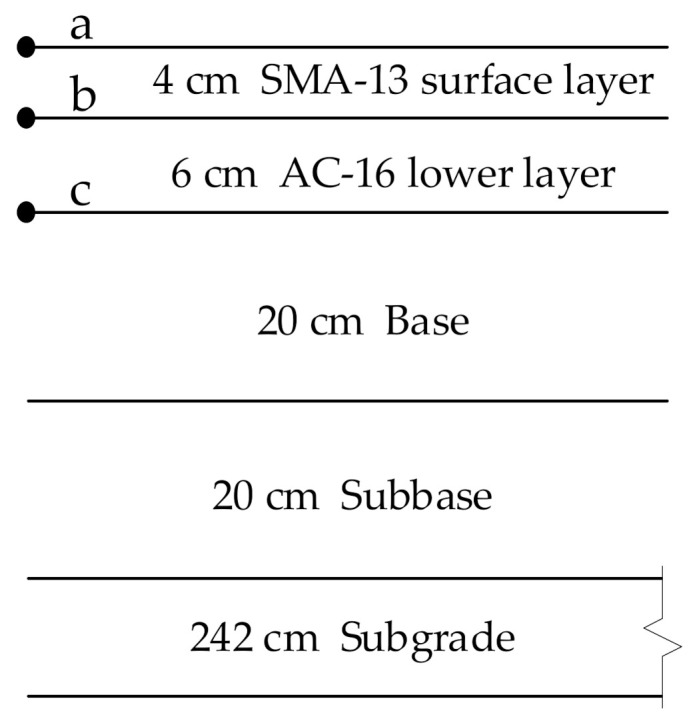
Pavement structure and analysis points. where a is the surface position of surface layer, b is the bottom position of surface layer, c is the bottom position of lower layer.

The full-scale model was established using the typical structural form commonly adopted by China’s first-class highway asphalt pavements, as shown in Figure 2. The surface layer was SMA-13, and the lower layer was AC-16. The void ratio of SMA-13 and AC-16 was 3.5% and 4%, respectively, and their voids were completely saturated. The two-dimensional model and analysis points of saturated asphalt pavement are presented in Figure 2. The points a, b, and c are located on the axisymmetric edge line, where a and b are points on the surface and middle of the surface layer, respectively, and c is a point in the middle of the lower layer.

The finite element model of the pavement structure is an axisymmetric model with a horizontal radius of 3 m and a depth of 2.92 m. The mesh element comprises a quadratic reduction integral element of 1200 elements. The asphalt mixture layer depends on the water phase transition effect of water icing, which is involved in the mesh ultra-refinement treatment. The other layers do not depend on the phase transition effect. Instead, they participate in the mesh refinement treatment. The axis of symmetry on the left side of the model is vertically free and laterally fixed constraints. The outer side of the right side of the model is also vertically free and laterally fixed constraints. The horizontal and vertical directions of the model’s bottom are fixed constraints. The pavement’s surface was the cooling boundary, the right and bottom edges of the pavement structure were the heat insulation boundary, and the soil foundation was at a constant temperature.

#### 2.3.2. Determination of the Material Parameters

(1) Nonwater phase transition

In the case of the nonwater phase transition, assuming that the voids of the asphalt mixtures are in a completely dry state, only the temperature shrinkage of the asphalt mixtures was considered. All of the mixture parameters are given in Table 1.

(2) Water phase transition

At the water phase transition, the voids were assumed to be completely saturated with water. The water is completely frozen at low temperatures. Therefore, the asphalt mixture was composed of the skeleton (hereinafter referred to as the matrix), water, and ice. Denoting the total volume of the asphalt mixture as V, and the volumes of the matrix, water, and ice as V_m_, V_w_, and V_i_, respectively, the volume percentage of these three parts θ_m_, θ_w_, θ_i_ were computed as follows:

(11) $$\theta_{m}=\frac{V_{m}}{V},\theta_{w}=\frac{V_{w}}{V},\theta_{i}=\frac{V_{i}}{V}$$

Which satisfied

(12) $$\theta_{m}+\theta_{w}=1,\theta_{s}+\theta_{i}=1$$

The density of the asphalt mixture before and after the water phase transition, respectively, was computed as:

(13) $$\rho_{m}=\theta_{s}\rho_{s}+\theta_{w}\rho_{w}$$

(14) $$\rho_{m}=\theta_{s}\rho_{s}+\theta_{i}\rho_{i}\;$$

where ρ_m_ is the density of the asphalt mixture, ρ_s_ is the density of the basic material, ρ_w_ is the density of water (1 g/cm^3^), and ρ_i_ is ice density (0.92 g/cm^3^).

Similarly, the asphalt mixture’s density, heat capacity, and thermal conductivity before and after the water phase transition were calculated using the abovementioned mixture theory. When the water in the voids of the asphalt mixture freezes and its phase state changes, the equivalent material parameters change abruptly. In this study, a piecewise function (Equation (15)) was used for establishing the relationship between the temperature and the density, specific heat, and thermal conductivity. Further, the step function in COMSOL was utilized for smoothing the mutant material parameters in the phase change interval (T_S_, T_E_).

(15) $$u_{A}\left(T-0\;{}^{\circ}C\right)=\left\{\begin{array}{l} A,\;\;\;\;\;T\leq 0\;{}^{\circ}C\;\\ B,\;\;\;\;\;\;T>0\;{}^{\circ}C \end{array}\right.$$

where u_A_ is the step function, and A and B are constants.

The equivalent temperature expansion coefficient considering the phase transition of water in the voids was computed using Equations (7) and (8). The temperature change had little effect on the coefficient of linear expansion of the matrix, which can be considered a constant. Therefore, the equivalent linear expansion coefficients, γ_w_ and γ_i_, outside the water phase transition interval could be calculated using the theory of mixtures, where the linear expansion coefficient of water is 4.3 × 10^−5^ (1/K) and the linear expansion coefficient of ice is 5.2 × 10^−6^ (1/K). The equivalent linear expansion coefficient in the water phase transition interval was calculated using Equation (8). The void ratios of SMA-13 and AC-16 were 3.5% and 4%, respectively. According to the relationship between volume and radius, the outer radius, b, of SMA-13 and AC-16 was 3.06a and 2.94a, respectively. Referring to the previous simulation of icing, the water phase transition temperature range of void water in asphalt mixture, the temperature interval of this paper was (T_S_, T_E_), where T_E_ = −0.5 °C, T_S_ = 0.5 °C [20]. Substituting all the parameters into Equations (7) and (8), the equivalent linear expansion coefficient, γ_wi_, of SAM-13, having a void ratio of 3.5% and AC-16 having a void ratio of 4%, when undergoing a water phase transition, were computed as −3.5 × 10^−4^ (1/K) and −4.5 × 10^−4^ (1/K). 

According to the measured dynamic modulus of the asphalt mixture at different temperatures, the modulus value of the asphalt mixture is related to its temperature [29,30,31]; the functional relationship between the dynamic modulus of asphalt mixture and temperature is obtained by fitting as follows.

The function providing the relationship between the dynamic modulus, *E*, MPa of AC-16, and the temperature change was given by:

(16) $$E=33.743\times 10^{3}\times e^{-0.073T}$$

where T is the temperature, °C; *E* is the dynamic modulus, MPa.

Similarly, the function providing the relationship between the dynamic modulus of SMA-13 and the temperature change was given by:

(17) $$E=33.487\times 10^{3}\times e^{-0.047T}$$

where T is the temperature, °C; *E* is the dynamic modulus, MPa.

In the lower temperature state, the dynamic modulus of the asphalt mixture is quite different from the elastic modulus of ice. Therefore, the dynamic modulus of the asphalt mixture was directly used as the overall modulus of the structure [16].

(3) Cooling conditions

The road surface cooling conditions were obtained by referring to the changes in the winter temperature measured on a certain day in Changchun City, Jilin Province. In this study, the temperature was simulated with a period of 18 h from 12:00 noon to 6:00 a.m. of the next day. The next day was simulated with a time span of 18 h. The temperature data were fitted by the following linear function:

(18) T=4.685−1.342t 

where *t* is the time, h; T is the temperature, °C.

The temperature of the model was initialized as 4.685 °C, and the road surface temperature dropped from 4.685 °C to −19.474 °C within 18 h.

#### 2.3.3. Solving Parameters

At the nonwater phase transition, the voids of the asphalt mixture are completely dry, and the material parameters do not change abruptly. Therefore, the transient solver adopted the automatic nonlinear Newton method in the full coupling mode, and the interval of the time step was set to 0.5 h. At the water phase transition, the asphalt mixture is completely saturated with water, and the water in the voids freezes; thus, undergoing a water phase transition results in a sudden significant change in the material parameters. To avoid the problem of highly nonlinear parameters, the automatic highly nonlinear Newton method in full coupling mode is adopted in the transient solver. In addition, to speed up the convergence of the model, the minimum damping coefficient was adopted in the solution process. The time step of the water phase transition interval was encrypted using a time interval of 0.01 h within the water phase transition range and a time interval of 0.5 h outside the water phase transition range.

## 3. Results and Discussion

The expansion of water in the asphalt mixture voids causes stress and deformation in freezing and thawing. In addition, the asphalt pavement structure also produces stress and deformation due to the temperature shrinkage effect at low temperatures. Therefore, it is essential to calculate the water phase transition of the asphalt mixture and nonwater phase transition states, and analyze their stress and strain change characteristics.

### 3.1. Temperature Comparison

**Figure 3:**
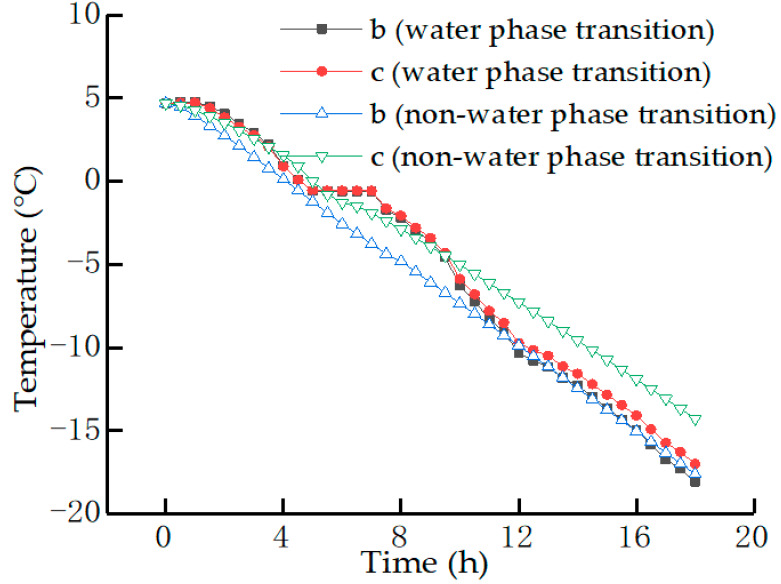
Varying characteristics of temperature with time.

The curves in Figure 3 demonstrate the temperature of the analyzed points b and c as a function of time. It can be observed that at the water phase transition, the internal temperature change of the pavement surface is nonlinear, whereas the temperature change is linear at the nonwater phase transition. The nonlinearity of temperature change at the water phase transition is due to the fact that when the internal temperature of the pavement surface drops to about 0 °C, the water freezes and releases latent heat, and thus, the temperature can be maintained for a period of time without dropping. This phenomenon does not occur at the nonwater phase transition. However, regardless of whether a phase change occurs or not, the temperature of the surface layer always lags behind that of the lower layer.

### 3.2. Change of Stress and Strain with Temperature

**Figure 4:**
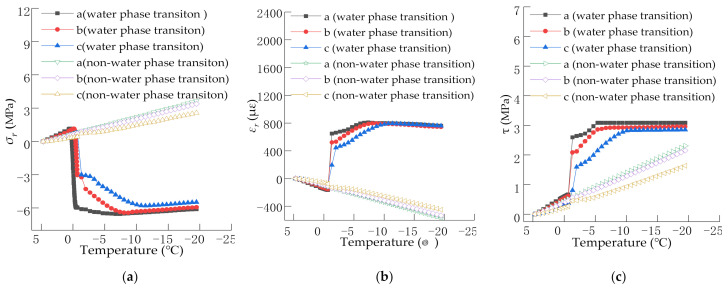
Varying characteristics of stress and strain with temperature, where (**a**) is the horizontal stress, (**b**) is the horizontal strain, and (**c**) is the shear stress.

It can be seen from Figure 4a that the horizontal stress at the analyzed points a to c is tensile stress, and their value changes little as the temperature decreases during the initial cooling. When the temperature drops to about 0 °C, the phase change of water occurs, and the stress immediately transforms from tensile stress to compressive stress. This is because the water in the voids of the asphalt mixture reaches the water phase transition point as the temperature drops to 0 °C, and thus freezes and expands. In contrast, the horizontal stress at the nonwater phase transition continues to increase linearly even though the temperature decreases and no stress mutation occurs. At the beginning of the phase transition of water in the voids, as the water freezes over time, the compressive stress continues to increase. After the temperature dropped to −10 °C, the compressive stress tended to become stable, and as the temperature dropped below −10 °C, the compressive stress slightly decreased. The maximum compressive stress at the analyzed point a is 6.54 MPa, whereas at the analyzed points b and c are 6.35 MPa and 5.79 MPa, respectively. The tensile stress at the nonwater phase transition tended to increase until the last moment of cooling, and the maximum value is 3.63 MPa, which also occurs on the surface of the surface layer.

It can be seen from Figure 4b that the horizontal strains at the analyzed points a to c are all compressive strains under both the water phase transition and the nonwater phase transition during the initial cooling. When the temperature drops to 0 °C, the horizontal strain during the water phase transition immediately transforms from compressive strain to tensile strain. However, the compressive strain at the nonwater phase transition continues to increase linearly with the decrease in temperature, and no strain mutation occurs. The maximum compressive strain is 570.42 µε. After the water in the voids freezes and expands, the tensile strain continues to increase with the freezing time. When T was –10 °C, the tensile strain tended to be stable, and the deformation no longer increased. Even with the continuous decrease in temperature, the tensile strain tended to decrease slightly. The maximum tensile strain is observed at point a, followed by point b, and the smallest at point c, with values of 809.22 µε, 796.09 µε, and 793.81 µε, respectively.

It can be seen from Figure 4c that although the shear stress at the water phase transition and the nonwater phase transition increases with the decrease in temperature, the change law is different. At the beginning of cooling, the shear stress at the water phase transition and the nonwater phase transition fluctuates linearly with a decrease in temperature. When the temperature reaches the water phase transition point, the shear stress during the water phase transition increases sharply. The shear stress continuously increased and tended to be stable at –10 °C with the decreased temperature. However, the shear stress during the nonwater phase transition increases linearly with the decrease in temperature. At the water phase transition, when the water in the voids freezes and expands, the shear stress at point a is the largest, followed by that at point b, and point c is the smallest, with the maximum values of 3.11, 2.91, and 2.28 MPa, respectively. The maximum shear stress at the nonwater phase transition is 2.31 MPa, which is 0.75 times that at the water phase transition.

### 3.3. Distribution Characteristics of Stress and Strain

**Figure 5:**
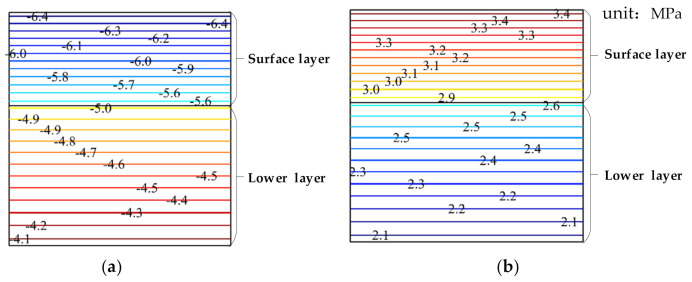
Cloud chart of the horizontal stress (in MPa) varying with the pavement depth; where (**a**) is the case of the water phase transition, and (**b**) is the case of the nonwater phase transition.

When T = −10 °C, the cloud chart of horizontal stress of pavement structure with depth will vary, as shown in Figure 5. The horizontal stress of different pavement depths is different according to the stress cloud chart. In general, the stress is greater when it is closer to the pavement surface, and thus the damage to the structure is also larger. Compared to the horizontal stress nephogram of the water phase transition and the nonwater phase transition, the horizontal stress of pavement structure is compressive stress. The internal horizontal stress of pavement structure at the nonwater phase transition is tensile stress, and the compressive stress generated by the water phase transition is greater than the tensile stress of the nonwater phase transition, and the compressive stress is about 1.8 times the tensile stress.

**Figure 6:**
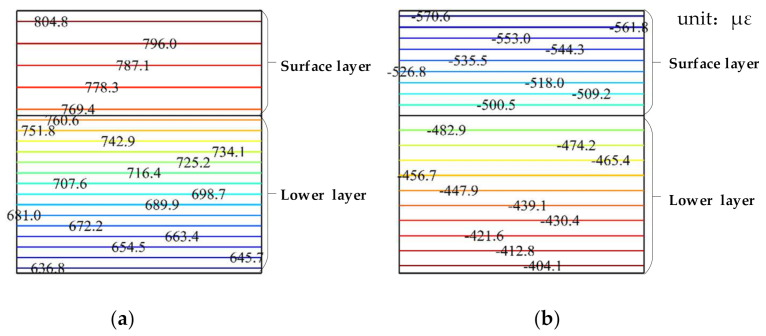
Cloud chart of horizontal strain variation with the pavement depth, where (**a**) is the case of water phase transition and (**b**) is the case of nonwater phase transition.

Figure 6 shows that the cloud chart of horizontal strain changes with a depth at −10 °C, and that the horizontal strain inside the pavement structure gradually decreases with the increase in depth. When the water in the voids freezes and expands, the pavement structure interior is in a state of tension, whereas it is in a state of compression during the nonwater phase transition. The deformation during the water phase transition is greater than that at the nonwater phase transition.

**Figure 7:**
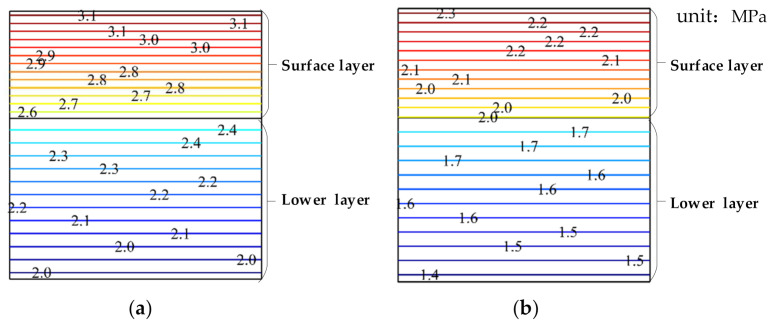
Cloud chart of the shear stress as a function of the pavement depth, where (**a**) is the case of the water phase transition and (**b**) is the case of the nonwater phase transition.

Figure 7 is the cloud chart of shear stress changing with pavement depth when T = −10 °C. It can be seen from Figure 7 that the various characteristics of shear stress with the pavement depth are consistent with that of the horizontal stress and horizontal strain with the pavement depth. The closer to the pavement surface, the greater the stress value. The shear stress under the water phase transition is larger than that under the nonwater phase transition.

### 3.4. Discussion

From the numerical simulation results presented above, it can be observed that the effect of the water phase transition during the freezing and thawing process of the asphalt mixture is more complicated than the nonwater phase transition effect. The pavement structure is under tension, resulting in large compressive stress and the appearance of shear inside the pavement structure. Therefore, the pavement structure is more prone to shear failure.

As shown in Figure 4, when the frost heave occurs in the asphalt mixture with a continuous decrease in temperature, the freezing time continues to extend, and the stress and strain continue to increase. This observation is the same as that seen in previous research results, which state that “the longer the freezing duration, the greater is the frost heave damage” [17]. After the temperature drops to –10 °C, the stress and strain tend to be stable, and as the temperature continues to decrease, the stress and strain no longer fluctuate. This result is also the same as that found in previous research, which states that “when the asphalt mixture is frozen for a long time, the damage no longer evolves” [19]. Therefore, the above analysis qualitatively verifies the rationality of the thermo-mechanical coupling model of asphalt mixtures.

Horizontal compressive stress is generated inside the pavement structure when the water in the voids of the asphalt mixture freezes and expands. Therefore, in this study, the compressive strength of a single axial compression test under a single freeze–thaw condition was used for verifying the numerical simulation results. The compressive strength under freeze–thaw conditions is different due to the difference in the design of the materials used in the asphalt mixture. In general, the compressive strength value of the uniaxial compression test measured by the test is about 4.5–6 MPa [32,33]. When frost heaving occurs in the asphalt pavement, the maximum horizontal compressive stress calculated by the numerical simulation ranges from 5.8 MPa to 6.5 MPa. The numerical simulation results are in good agreement with the experimental results. Due to the variability of the material types and parameters, the above results are somewhat different. However, generally in the same grade of magnitude, they also support the rationality of the calculation results in this study from a quantitative perspective.

This study considers the frost heaving of asphalt mixture, but does not consider the freeze–thaw damage. The relevant experimental research shows that the internal stress caused by the freezing of the water in the voids in the asphalt mixture leads to internal microcrack damage that continues to expand during the freeze–thaw cycle [2]. Therefore, damage due to the frost-heave effect on the asphalt mixture can be regarded as a type of fatigue damage. Because the fatigue damage mechanism is very complex, this study did not consider the damage of the asphalt mixture during the water phase transition. A subsequent study will consider the influence of the freeze–thaw cycle on the structural damage of the asphalt mixture, and a simulation of the increase in the number of voids in the process of the freeze–thaw cycle will be performed. In addition, the expansion coefficient of the asphalt mixture calculated in this study should be verified and corrected by a method in future work.

## 4. Conclusions and Recommendations

Some conclusions from this study can be drawn:

(1) The frost-heaving effect on the asphalt mixture caused by the ice–water phase transition cannot be ignored. When the water in the voids is completely frozen, the volume of the mixture changes suddenly, and the corresponding equivalent temperature expansion coefficient is approximately 20 times the nonfrost-heaving expansion coefficient. Frost heaving results in large compressive stress, shear stress, and expansion strain on the surface and inside the pavement structure. In addition, the pavement surface is the most unfavorable position for frost heaving.
(2) The calculation results presented in this paper are consistent with previous research results from a quantitative and qualitative perspective. It has been proven that the frost-heaving stress continues to increase as the freezing time increases. When the frost heaving is complete, the frost-heaving stress tends to be stable, and the compressive stress generated by frost heaving is equivalent to the low-temperature compressive strength of the asphalt mixture. The frost-heaving effect simulation method established in this study can avoid the strict meso-modeling process and efficiently calculate the frost-heaving effect in the pavement structure.
(3) This study only considers the single frost-heaving effect on the asphalt mixture in the process of the freeze–thaw cycle. To describe the freeze–thaw failure effect of the asphalt mixture more truly in this paper, the equivalent temperature expansion coefficient of frost heaving was verified by experiments. On the other hand, the failure effect of asphalt mixture in multiple freeze–thaw cycles is simulated.
(4) This study is of great significance for accurately evaluating the frost resistance of asphalt pavement and provides a theoretical basis for the frost resistance design of asphalt pavement. In this paper, only a single frost-heaving effect of asphalt mixture was considered, and the effect of multiple freeze–thaw cycles was not considered. This study provides ideas and references for a follow-up study on the macro frost-heaving damage of asphalt pavement.

## Figures and Tables

**Table 1:** The material parameters.

	Material Type	SMA-13	AC-16	Base	Subbase
Parameter Name					
Density (g/cm^3^)		2.44	2.43	2.2	2.1
Specific heat capacity(J/(kg.K))		768	817	942.9	942.9
Thermal conductivity (W/(m.K))		1.30	1.25	2.2	2.2
Coefficient of linear expansion (1/K)		1 × 10^−6^	15 × 10^−6^	\	\

## Data Availability

All of the data used to support the results of this study can be obtained from the corresponding authors according to the requirements.
